# Detection of small bunches of ions using image charges

**DOI:** 10.1038/s41598-018-28167-6

**Published:** 2018-06-28

**Authors:** Paul Räcke, Daniel Spemann, Jürgen W. Gerlach, Bernd Rauschenbach, Jan Meijer

**Affiliations:** 1 Universität Leipzig, Felix Bloch Institute for Solid State Physics, Linnéstr. 5, 04103 Leipzig, Germany; 2 Leibniz Joint Lab “Single Ion Implantation”, Permoserstr. 15, 04318 Leipzig, Germany; 3 Leibniz Institute of Surface Engineering (IOM), Permoserstr. 15, 04318 Leipzig, Germany

## Abstract

A concept for detection of charged particles in a single fly-by, e.g. within an ion optical system for deterministic implantation, is presented. It is based on recording the image charge signal of ions moving through a detector, comprising a set of cylindrical electrodes. This work describes theoretical and practical aspects of image charge detection (ICD) and detector design and its application in the context of real time ion detection. It is shown how false positive detections are excluded reliably, although the signal-to-noise ratio is far too low for time-domain analysis. This is achieved by applying a signal threshold detection scheme in the frequency domain, which - complemented by the development of specialised low-noise preamplifier electronics - will be the key to developing single ion image charge detection for deterministic implantation.

## Introduction

Ion implantation is widely used for decades, for example in semiconductor technology, and has recently enabled groundbreaking magnetometry and qubit experiments with e.g. NV-centres in diamond^1–5^ or single phosphorous atom devices in silicon^6–11^. For the fabrication of scalable quantum devices employing single atoms^12^, but also for semiconductor doping on the smallest scale, controlling the exact number of the implanted ions is decisive for device performance^13^.

Different approaches are pursued to realise counting a small number of single ions during implantation, but so far success is very limited^14^. The developed techniques can be categorised in pre-detection and post-detection schemes with regard to the implantation event. Whereas in a post-detection scheme, secondary processes initiated by the ion implantation are used for ion detection, a pre-detection scheme is characterized by an ion detection prior implantation. The significant advantage is that detection efficiencies <100% can be allowed by simply discarding all undetected ions from implantation.

The most prominent pre-detection approach is to store a single cooled ion in a Paul trap and accelerate it towards the sample, after its presence in the trap has been detected optically^15,16^. The main drawbacks, however, are the significant experimental effort and slow implantation rate. For post-detection, either secondary electrons^13,17^ or the induced charge of the impacting ion (IBIC)^7,14^ are used as registration signal. For secondary electron detection and IBIC, the signal increases with the kinetic energy of the implanted ions, limiting the sensitivity for low ion energies. However, for a spatially highly precise ion placement, low kinetic energies are desirable, e.g. <5 keV for P in Si, to reduce ion straggling. Up to now, ion beam induced charge measurements have been demonstrated for P implantation in silicon with ion energies down to 14 keV^7,14^. As the sample has to serve as a detector in case of IBIC, it will be difficult to apply induced charge detection to arbitrary sample materials and depending on the material system and ion type, there is always a minimum kinetic energy required for successful detection. Pacheco and co-workers, for example, achieved a detection efficiency of 87% for 20 keV Sb ion implantation into a silicon based device^18^. In comparison, in image charge detection, a pre-detection scheme, the sensitivity only depends on the number of charges to be detected, so that at very low ion kinetic energies it has the potential of becoming the only viable detection scheme.

The ultimate goal is to devise a method that allows deterministic implantation of any ion species - in the extreme case a single ion - with nanometre resolution into arbitrary samples with high throughput. Besides limitations to specific materials and ions, every post-detection method necessarily has to guarantee a detection efficiency of 100% for single ion implantation, while at the same time no false positives are allowed. It is shown below, that with a dedicated pre-detection scheme, a detection efficiency smaller than 100% does not impair the feasibility of the method, while under this condition, false positive detection events can be avoided much easier.

The approach presented in this work is based on the signal induction by the image charge of a moving ion^19^. The fact that moving charges can induce a measurable image charge current was noticed in the 1930s and has been described theoretically by Shockley and Ramo^20,21^. The idea of the presented detection scheme is based on results in the mass spectrometry of large, slow molecules carrying a high number of net charges.

When a moving charge passes a single electrode, the charge signal induced in that electrode is a peak in the time domain. In image charge mass spectrometry, ions are commonly trapped and cycled next to, or through a single hollow electrode using a dedicated ion optical arrangement, so that a periodic signal can be recorded and analysed. In terms of the signal-to-noise ratio (SNR), repeating a measurement *N* times improves the sensitivity by $\mathrm{1/}\sqrt{N}$ for uncorrelated noise. This means that by increasing the trapping time or equivalently the number of cycles through the electrode, the sensitivity is improved. This is also equivalent to decreasing the frequency bandwidth of the recorded periodic signal.

Benner demonstrated such a measurement with a detection limit of 250 elementary charges (*e*)^22^. By increasing the trapping time to 3 s, i.e. decreasing the bandwidth, the detection limit was decreased to 7*e* by Jarrold and co-workers^23–26^.

For implantation at the nanoscale, ions have to be detected on their linear trajectory on the way to the sample. Any other, more complicated trajectory, for example cycling ions for longer measurement duration, is incompatible with the high demand, i.e. the low acceptance, of the ion optical system required for spatially precise implantation. A linear array of electrodes offers the possibility to create a periodic signal in a single ion pass. Experimental results based on linear detector arrays have been demonstrated as well^27–29^. As long as the signal-to-noise ratio permits, mass and charge can be extracted from the time-of-flight and amplitude of the signal. For example, Smith *et al*. used autocorrelation analysis of recorded raw data to extract this information, although the signal did not in general extend significantly above the noise floor^28^.

Theoretically, the length of such a linear array can be scaled arbitrarily to reach a detection limit of 1*e*, if the signal analysis can be carried out in the frequency domain^27^. In practice, however, the length is limited, for example, due to ion beam divergence.

In comparison to previous work on mass spectrometry, the image charge detector (ICD) envisaged for ion implantation works in a completely different kinetic energy regime, i.e. the ions possess much faster velocities, much lower mass and generally a lower number of charges per ion. Furthermore, in contrast to the literature reviewed, real time signal analysis methods need to be explored that enable a proper pre-detection of the ion on its way to the sample. On the other hand, ion implantation has the decisive advantage that all parameters of the ions to be detected are known, whereas mass spectrometry has the aim of measuring unknowns.

In this paper, the concept and optimal conditions for the application of image charge detection for deterministic implantation are explored. After a brief summary and review of image charge detector theory, our experimental results are presented, followed by a discussion of the possibilities for signal processing to realise a fast trigger output in case of successful detection. We believe that the presented study provides the starting point for the realisation of a single ion image charge detector.

## Theoretical Considerations

First theoretical considerations about the instantaneous currents induced by moving electrons were presented by Shockley and Ramo^20,21^. For an arbitrary arrangement of moving charges and grounded electrodes, they derived the expression

1 $${q}_{i}=-\,e{{\rm{\Phi }}}_{i}({\bf{r}})$$

for the charge *q*_*i*_ induced in a given electrode by a unit charge *e* at location **r**. The so called weighting potential Φ_i_ is calculated by setting the respective electrode to unit potential (*V* = 1, dimensionless) and all other electrodes to ground. If the point charge is moving with velocity **v** = d**r**/d*t*, it follows for the induced current

2 $${I}_{i}=\frac{{\rm{d}}{q}_{i}}{{\rm{d}}t}=-\,e\nabla {{\rm{\Phi }}}_{i}({\bf{r}})\cdot \frac{{\rm{d}}{\bf{r}}}{{\rm{d}}t}=e{{\bf{E}}}_{{\rm{i}}}({\bf{r}})\cdot {\bf{v}}\mathrm{.}$$

Here, the weighting field **E**_i_ has the unit m^−1^. The original derivations all refer to electrons, but if one considers an ion with more than one unit of charge, the elementary charge *e* in Eq. 2 is only multiplied by the number of charges.

**Figure 1:**
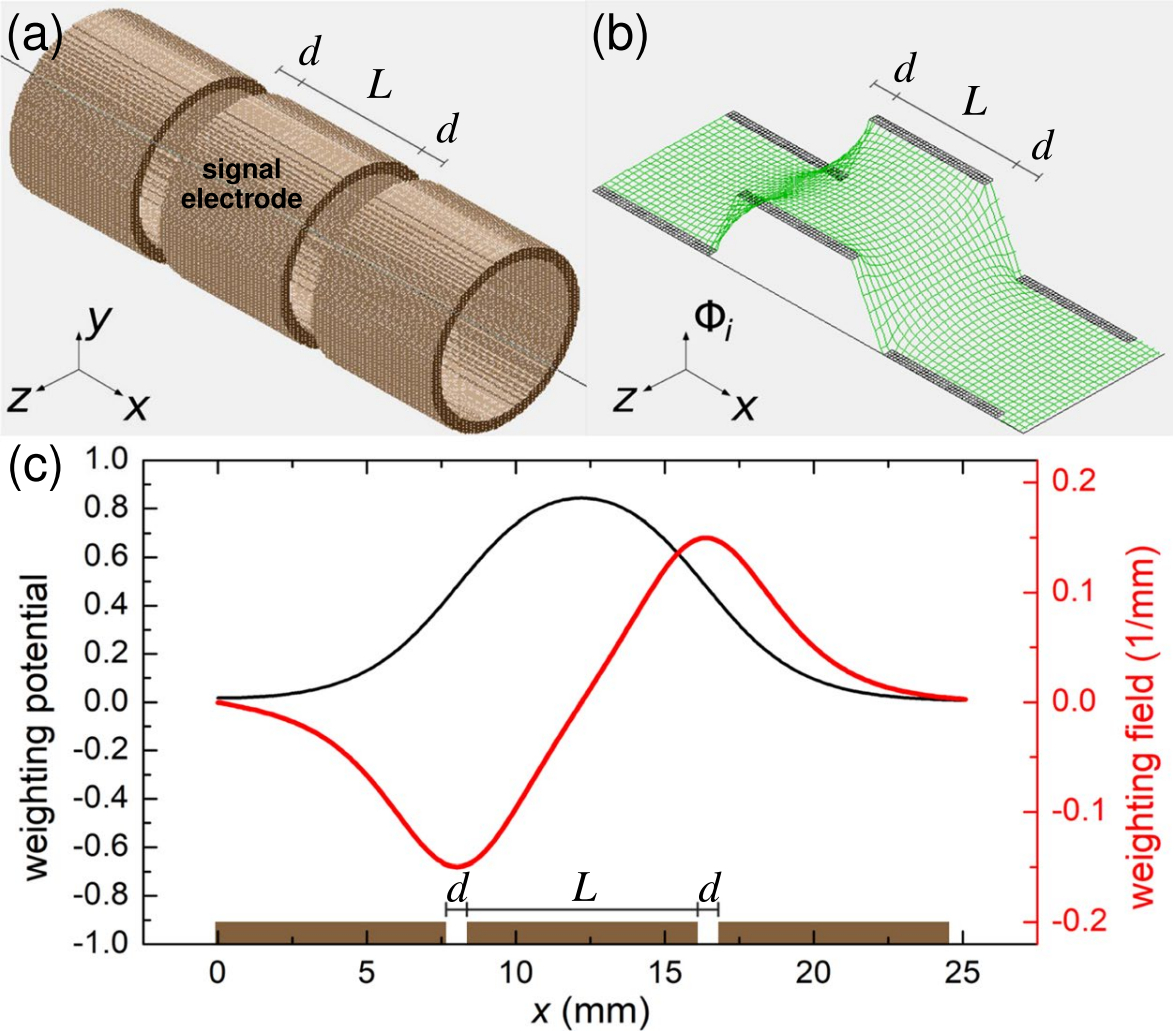
**(a)** Three cylindrical electrodes with symmetry axis as ion trajectory. *L* is the length of the central electrode, *d* is the spacing between the electrodes. **(b)** SIMION simulation of the weighting potential Φ_i_ associated with the central electrode, ranging from 0 to a maximum value of 1. **(c)** Weighting potential and weighting field along the symmetry axis, i.e. the ion trajectory.

For demonstration, an ion is considered on a straight trajectory, travelling through an arrangement of three cylindrical electrodes, similar to an einzel lens (see Fig. 1a). The weighting potential is calculated with SIMION^30^ by setting the central electrode to unit potential (Fig. 1b). The potential along the ion trajectory is shown in Fig. 1c, together with the weighting field, i.e. its spatial derivative. In this example, the weighting potential reaches a value of about 0.9 at the centre of the electrode. This means that the maximum induced image charge is 0.9 times the charge of the ion. This factor and the distribution of the potential solely depend on the geometry of the electrode arrangement. As stated above in Eq. 2, the induced current is proportional both to the weighting field (red curve in Fig. 1c) and the ion velocity in the direction of this field. In addition, when measured, the signal formed in an electrical circuit connected to such an electrode will also depend on the type and characteristics of amplification.

A transimpedance preamplifier produces a voltage signal proportional to the instantaneous current. Thus, the signal shape and amplitude are directly linked to the weighting field. For the latter to be increased, the electrode spacing would have to be reduced to achieve a higher gradient in the potential. However, this cannot be taken to extremes, because in practice, the rise time of the preamplifier is a strict limitation.

The signal of a charge sensitive preamplifier, having a capacitor *C*_f_ as feedback component parallel to an operational amplifier, will be directly proportional to the weighting potential. The sensitivity is given by *C*_f_ and the image charge *q*_i_, such that the output voltage is

3 $${V}_{{\rm{out}}}=\frac{{q}_{{\rm{i}}}}{{C}_{{\rm{f}}}}\mathrm{.}$$

However, there are important restrictions. As the gain of the preamplifier is only given by *C*_f_ and the total input noise is amplified as well, the optimum signal-to-noise ratio depends both on the noise characteristics of the preamplifier input and the total capacitance at the input.

A voltage preamplifier directly reacts to the voltage in the electrodes, which results from the image charge and the total capacitance comprising the capacitance of the electrode with respect to ground and the input capacitance of the preamplifier electronics, given by

4 $${V}_{{\rm{out}}}=\frac{{q}_{{\rm{i}}}}{{C}_{{\rm{total}}}}\mathrm{.}$$

If the electrode structure outlined so far is repeated in a linear array so that every even electrode is connected to the preamplifier input and the odd electrodes in between are grounded, an alternating signal results from a single ion passage. The geometry of the electrode array can be adjusted to yield a sinusoidal signal shape which effectively results in an AC voltage pulse with the finite duration determined by the ion velocity and the electrode array length. The amount of energy in the fundamental Fourier component and the bandwidth, given by the inverse of the duration of the signal, determine how well it can be recovered from noise.

## Experimental Methods

**Figure 2:**
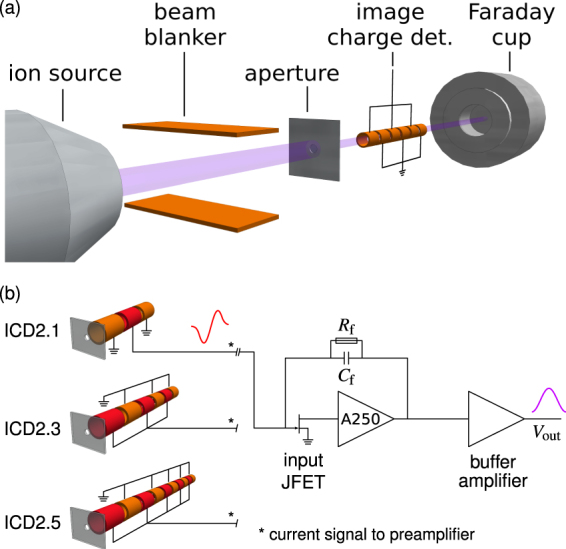
**(a)** Schematic of the experimental set-up to investigate image charge signals induced by low energy ion bunches. **(b)** Schematic depiction of the three different detector configurations used, ICD2.1, ICD2.3, ICD2.5, with one, three and five signal electrodes (marked in red), respectively. Exemplary, the transformation of the current signal (red) from detector version ICD2.1 into *V*_out_ through the A250CF preamplifier electronics is shown^31^.

A schematic of the set-up for image charge detection measurements is shown in Fig. 2. The whole set-up is mounted in a vacuum chamber evacuated to a base pressure of 1 × 10^−6^ mbar. An ion beam is created and focussed by a SPECS ion source ionising Argon, Nitrogen or other gases with thermally emitted electrons. Ions are accelerated to kinetic energies in the range from 15 keV and selected using a Wien filter. The beam blanker is controlled with a fast high voltage switch with a maximum potential of 500 V. In order to study the charge sensitivity of the detection set-up, ion bunches of sufficiently short duration are used to mimic ions of high charge state. For this purpose, rectangular voltage pulses are employed to form and transmit bunches of ions through the aperture. The rise time of the actual potential at the beam blanker plate was verified to be <25 ns.

From the aperture system, the ion bunches move through the image charge detector (ICD) and into the Faraday cup (FC) used to measure the ion beam current with a Keithley 6485 Picoammeter. All cables inside and outside the chamber are shielded.

The ICD signal is preamplified and analysed with a Rohde & Schwarz RTO2024 digital oscilloscope. In this work, two ICD systems were designed and studied. The first one (ICD1) is based on the Amptek A250 charge sensitive preamplifier and the second one (ICD2) uses the A250CF version of the preamplifier^31,32^. The results and discussion in this paper are focussed on the ICD2 set-up. Technical information about ICD1 and some results can be found in the supplementary material. In the ICD2 version, the preamplifier electronics is included in the A250CF package outside the chamber, connected to the detector array by BNC cables. The A250CF has a Peltier cooled input transistor and its feedback components are *C*_f_ = 0.5 pF and *R*_f_ = 1 GΩ. A buffer amplifier stage is built in between preamplifier and output (see Fig. 2b). Due to these specifications, ICD2 shows superior noise performance compared to ICD1. At the same time, it is less susceptible to electromagnetic interference problems, for example, from the beam blanker system, because it is placed outside the chamber.

Within the ICD2 set-up, the number of electrodes and their lengths can be varied flexibly for the different types of experiments presented here, where the electrodes connected to the preamplifier are referred to as signal electrodes. All electrodes are made of copper and have a length of *L* = 8 mm, an inner diameter of 3.8 mm and an outer diameter of 5 mm. Neighbouring electrodes are separated by *d* = 2 mm insulating spacers. At first, for the charge calibration measurements, a single signal electrode is used (ICD2.1). For the formation of a periodic signal, several signal electrodes are used with every second electrode being a signal electrode and the separating electrodes being grounded. Hence, the maximum number of signal electrodes is five (ICD2.5), because in total ten electrodes can be accomodated into the electrode support of ICD2. In Fig. 2b, the three different electrode arrangements used are shown (without insulating spacers and holders). The schematic shows the simplified preamplifier electronics as it is connected to the signal electrodes in each case. The red curve at ICD2.1 is the current signal of an ion bunch travelling through the single signal electrode. The integrating circuit of the A250CF charge-sensitive preamplifier transforms it into the indicated voltage pulse *V*_out_. The detailed signal shape, however, depends also on the length of the ion bunch. For more than one signal electrode the voltage pulse is repeated, forming a periodic signal which can be similar to a sinusoidal signal for ion bunches much shorter than the length of an electrode. Notice, that due to the integration of the two opposite current pulses of the ions going in and out of each signal electrode, the total induced charge is zero. Therefore, there is no exponential decay of the output voltage pulse as observed in standard photon or particle detection using similar preamplification. Also the rise-time of the preamplifier, which is in the order of 10 ns is no limitation to the measurements presented.

### Data availability

The datasets generated and analysed in this work are available from the corresponding author on reasonable request.

## Results

In order to evaluate the functionality of the electronic system comprised of the detector electrodes, preamplifier and their connections, different quantitative and qualitative measurements are necessary. First, a single electrode (ICD2.1) is used to determine the calibration factor of output voltage *V*_out_ to input number of charges. Then, the possibilities of combining a number of electrodes (ICD2.3, ICD2.5) at the input of a single preamplifier stage is explored, together with noise measurements and considerations of signal processing and real time analysis.

### Ion beam current calibration

At E_beam_ = 5 keV, the velocity of Argon ions with an atomic mass of 40 u is *v*_i_ = 1.55 × 10^5^ m/s, and so an electrode with L = 8 mm is traversed in ${t}_{{\rm{E}}}\approx 50\,ns$. To calibrate the ICD response to the number of charges inside an electrode, long pulses (*t*_pulse_ > *t*_E_) are used with a single signal electrode mounted and connected to the input of the preamplifier (ICD2.1). In this way, the electrode’s weighting potential is completely filled with a uniform distribution of charges and its integral yields the total effective image charge (similar reasoning was used in ref.^33^)

5 $${Q}_{{\rm{icd}}}=\frac{{I}_{{\rm{beam}}}}{{v}_{{\rm{i}}}}{\int }_{-\infty }^{\infty }{{\rm{\Phi }}}_{{\rm{i}}}(x){\rm{d}}x\mathrm{.}$$

**Figure 3:**
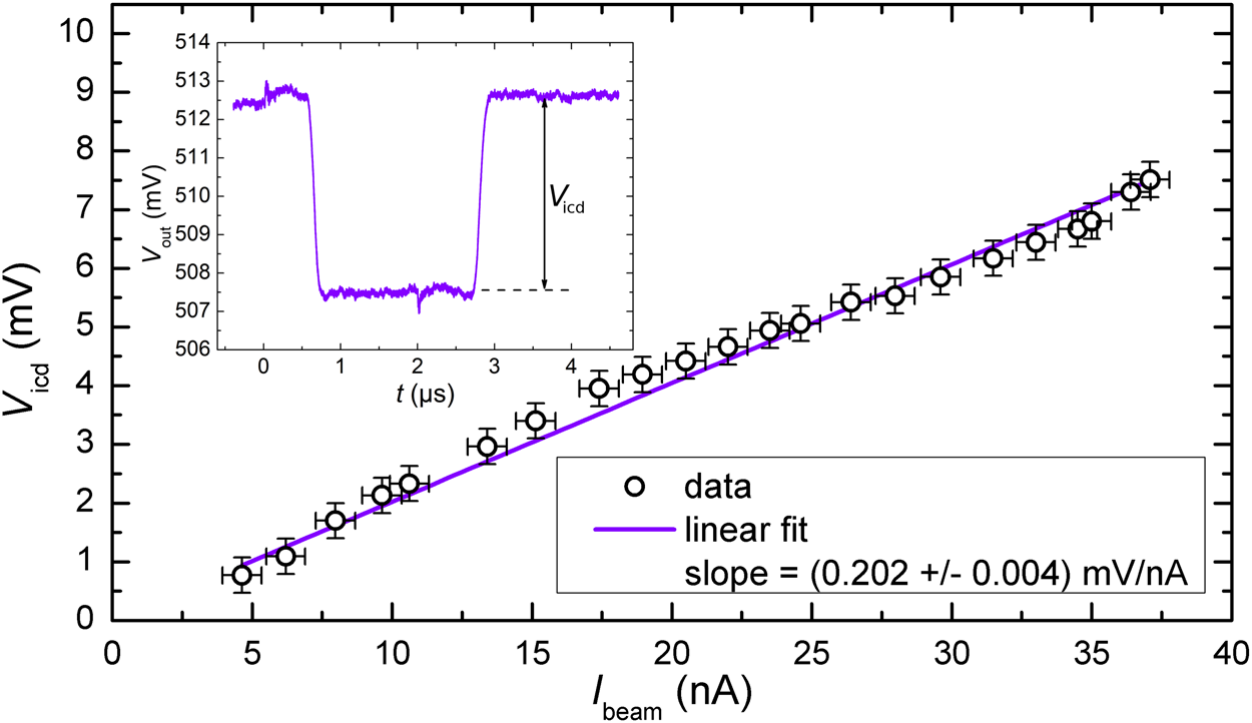
Linear ion beam current calibration curve for a single 8 mm signal electrode (ICD2.1). The inset shows a typical pulse waveform for *t*_pulse_ > *t*_E_ with *V*_icd_ determined as the signal pulse height.

To measure the ion beam current accurately and simultaneously during calibration, anti-pulses are used, i.e. the continuous beam is interrupted for a duration *t*_pulse_ > *t*_E_, as only the step height between “beam on” and “off” is of interest. The interrupting anti-pulse is repeated with a frequency of 1 kHz. Thus, the total average current is only lowered by a factor of 2 in 1000 for a 2 µs long anti-pulse, and quick averaging of 100 single acquisitions is possible. Figure 3 shows the linear calibration of the output voltage as a function of ion beam current for ICD2. The notation *V*_out_ refers to the voltage as a function of time as measured by the oscilloscope, while *V*_icd_ is the height of the signal plateau as indicated in the graph. A typical result from averaging 100 pulses to determine the signal height *V*_icd_ is shown in Fig. 3 (inset). The signal plateau waveform is superimposed with statistical and systematic noise contributions. The two high frequency pulses at 0 µs and 2.0 µs are interferences from the fast switching of the beam blanker. The time between the first switching (at 0 µs) and the falling edge of the signal (at 0.7 µs) is the time of ion travel from beam blanker to the ICD signal electrode. The calibration curve in Fig. 3 is shown with its linear fit. Due to a dominant low frequency statistical noise component, the calibration was affected by systematic averaging errors introduced by an unstable baseline. To counteract, the number of averaged waveforms was lowered in some cases which explains the observed deviations from perfect linearity. This low-frequency noise is not expected though to impair the detection of individual ion bunches in the following.

With the slope *V*_icd_/*I*_beam_ determined from the fit, the experimental sensitivity *S* can be calculated using Eqs 5 and 6.

6 $$S=\frac{{V}_{{\rm{icd}}}/{I}_{{\rm{beam}}}}{{Q}_{{\rm{icd}}}/{I}_{{\rm{beam}}}}\mathrm{.}$$

For ICD2.1, with ${\int }_{-16{\rm{mm}}}^{84{\rm{mm}}}{\rm{\Phi }}(x){\rm{d}}x=9.94\,{\rm{mm}}$, a sensitivity of *S* = 3.16 V/pC is calculated. The integral corresponds to the integral in Eq. 5 and is determined along a trajectory coinciding with the ion optical axis from numerical SIMION simulations using the actual electrode geometries. The integration boundaries are given with respect to the centre of the electrode and limited by the size of the simulation.

Nominally, the ICD2 preamplifier electronics has a sensitivity of 4.0 V/pC. The theoretical value is not reached in the experiment, which is attributed to uncertainties in the electronic components and impedance mismatches along the signal path. Nevertheless, the calibration is very well reproducible as long as the set-up is not changed and it is valid for all ICD2 configurations.

### Time domain detection

**Figure 4:**
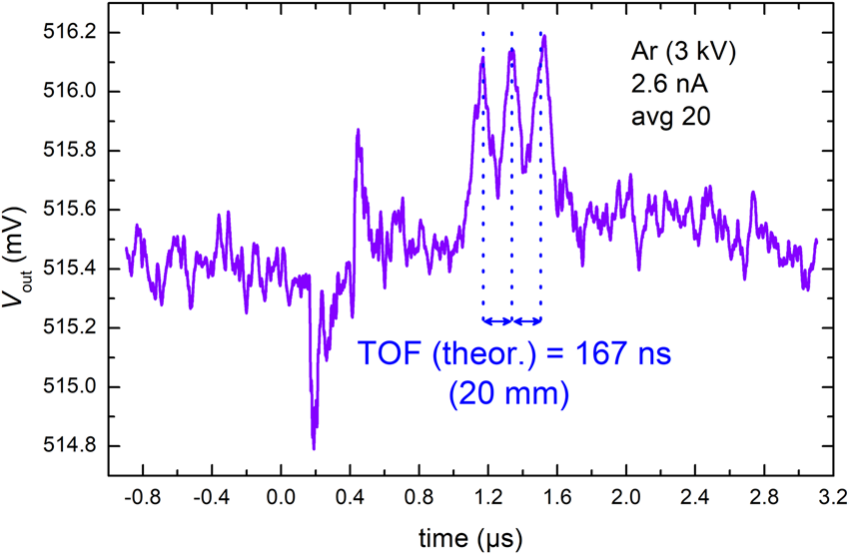
Oscilloscope output of averaging 20 single acquisitions of an ion bunch passage through three 8 mm signal electrodes in the ICD2 configuration. The annotated time-of-flight intervals are theoretically calculated from the ion beam energy of *E*_beam_ = 3 keV and the distance between the signal electrode centres, i.e. the period of the array of 20 mm.

If *n* electrodes are connected to the preamplifier input (compare Fig. 2b), the profile of the weighting potential should effect *n* peaks in the time domain signal, in case the ion bunch is shorter than the distance between two signal electrodes. Experimental confirmation for three electrodes (ICD2.3) is shown in the graph in Fig. 4 for ion bunches produced from a 3 keV Ar^+^ ion beam of 2.6 nA current. The time-of-flight between the electrodes is in agreement with calculation. Further measurements using two signal electrodes to determine the time of flight for different ion species and kinetic energies can be found in the supplementary material.

### Frequency domain detection and noise

**Figure 5:**
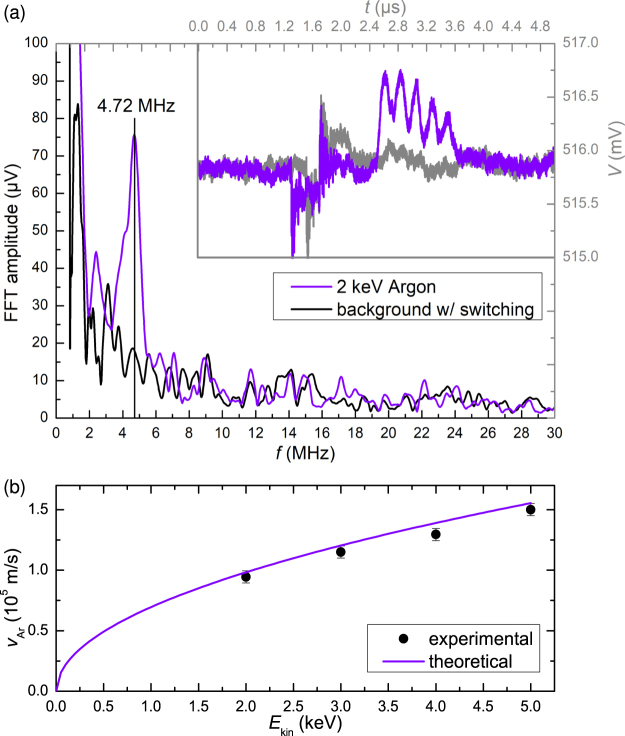
Fourier analysis of time signals recorded with five signal electrodes (ICD2.5). **(a)** Time signal (upper right, blue line, grey scale) and its FFT spectrum (bottom left, blue line, black scale) for 2 keV Argon ion bunches, shown together with the background spectrum (black/grey line) without ion bunch, but including switching interference, averaged over 100 acquisitions. **(b)** Ion velocity calculated from experimental FFT spectra for Argon ions with *m* = 40 u and 20 mm periodic length, dependent on ion beam energy and theoretical curve from $v=\sqrt{2{E}_{{\rm{kin}}}/m}$.

**Figure 6:**
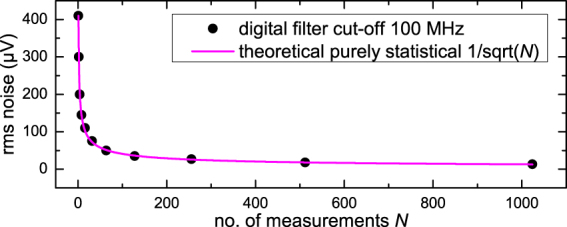
Rms noise voltage of ICD2 averaged over *N* acquisitions, excluding the interference from switching the beam blanker. The theoretical $\mathrm{1/}\sqrt{N}$-dependence expected for uncorrelated statistical noise is shown for comparison.

For frequency domain analysis, the maximum number of five signal electrodes was used (ICD2.5). Small 2 keV argon bunches produced from a 2.5 nA beam current were sent through the electrodes. The resulting periodic signal can easily be transformed into an FFT spectrum, as demonstrated in Fig. 5a. In this case, the time trace is recorded with full bandwidth and the FFT is performed live with the oscilloscope. Despite the included switching interferences and the short signal pulse, a clear peak rises above the background spectrum at 4.72 MHz. The velocity of the ions is calculated from the peak frequency and the known electrode arrangement, i.e. 20 mm periodic length. Taking into account the acceleration voltage, the ion mass can be calculated, making this experiment effectively a mass spectrometry measurement. Calculated velocities of ions with different kinetic energies are plotted in comparison with nominal values in Fig. 5b. Good agreement is demonstrated, but the fact that all values are slightly lower than the calculated ones points to a yet unknown systematic error in the set-up. Possible sources of this error are the reliability of setting the accelerating potential in the ion source, the Wien filter settings and probably also the FFT algorithm used. The sensitivity, that is the minimum number of charges detectable, depends on the noise of the preamplifier electronic system which includes the detector electrodes. Noise measurements of the two set-ups ICD1 and ICD2 reveal the differences in noise characteristics under different operating conditions. Measurements and explanations regarding ICD1 can be found in the supplementary material. The data from ICD2 is shown in Fig. 6. A digital 100 MHz low-pass filter was applied. The root-mean-square (rms) noise voltage as a function of the number *N* of individual acquisitions used for averaging follows the theoretically predicted statistical behaviour over a wide range of *N* (Fig. 6). This proves that significant contributions from systematic noise sources and interferences have been excluded by optimising the system and digital filtering.

The frequency domain opens up the possibility to recover a signal from noise. Whereas in the time domain, a signal becomes recognisable only if it significantly exceeds the total rms noise level (noise floor), in frequency analysis, the SNR can be given as a function of the spectral noise density *ν*_rms_ and the fundamental frequency component *V*_peak_, together with its bandwidth Δ*f* which results from the finite signal duration. Consequently, it can be taken as

7 $${\rm{SNR}}=\frac{{V}_{{\rm{peak}}}}{\sqrt{{\rm{\Delta }}f}{v}_{{\rm{rms}}}}\mathrm{.}$$

If the expected signal frequency is known, the existence of the signal can be probed in the Fourier spectrum, by setting a suitable threshold on a narrow interval around the expected frequency. If this threshold is exceeded, an ion or ion bunch is considered detected. In a deterministic single ion implantation process, the ion is permitted on its path to the sample only when the existence of the expected detector signal is registered (detection).

**Figure 7:**
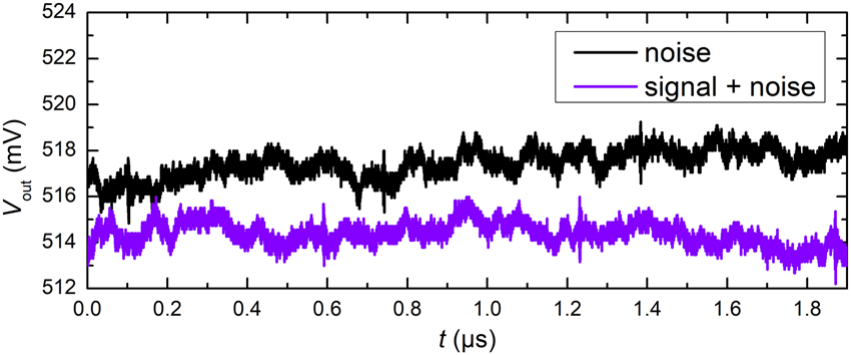
Pure noise and signal with noise; raw data examples without acquisition averaging. The rms noise level is approx. 600 µV, corresponding to 1200*e*. Switching interferences are chosen to lie outside (before) the region of interest.

This detection scheme has been tested for 4 keV Argon ion beam bunches in the live Fourier spectrum of single acquisitions using ICD2.5. Before the measurement, the ion beam current was lowered, so that a single bunch comprised approximately 300 ions, i.e. 300 elementary charges. In principle, the number of ions in each bunch might be subject to variations, as it cannot be measured simultaneously. The raw data shows that it is hardly possible to distinguish signal from noise in the time domain (Fig. 7). The number of detections divided by the total of 10^4^ acquisitions which all contain an ion bunch signal is called detection efficiency or rate (or true positive rate). In order to determine the false positive rate, the beam is switched off. Now, none of the acquisitions contains an ICD signal and the number of false detections is counted. The successful detection and false positive rates for different thresholds set in the Fourier spectrum are given in Table 1. As can be seen, increasing the threshold reduces the false positive rate very effectively with an acceptable reduction of the detection rate. In a deterministic ion implantation experiment, low false positive rates are essential for laying down error-free arrays of single atoms. Here, a detection rate of, for example, 50 means that on average every second ion is discarded by a beam blanker downstream of the ICD and not implanted, because the ion was not successfully detected with the ICD. Effectively, a lower detection rate only results in a reduced implantation rate.

**Table 1:** Detection and false positive rates for different thresholds set in the live FFT of single acquisitions with and without ion bunches, respectively.

Threshold (μV)	Detection rate (%)	False positive rate (%)
150	92	1.7
175	76	0.3
200	51	0.05
225	26	0 in 10^4^

## Discussion

In the current set-up, specific limitations inhibit the demonstration of a lower detection limit for the charge of an ion bunch. These include the method of bunch formation with an electric blanker, limited Wien filter accuracy, ion beam divergence and instabilities at very low beam currents. It can clearly be seen, that because of this, the extracted signal deviates significantly from a sinusoidal waveform with constant amplitude (see Fig. 5a). Therefore, the total signal power is not solely associated with the fundamental frequency given by velocity and electrode period. This affects the ability to detect in Fourier space. For example, the noise floor in the spectrum in Fig. 5a corresponds to less than 20 µV or 40*e*, but multiples of this number of charges are needed for observation, because the main Fourier peak carries only part of the signal. Furthermore, the form of the signal from a bunch of ions is determined by the convolution of weighting potential and ion distribution. However, when considering single ions instead of bunches, an ideal geometry for creating sinusoidal signals can be constructed easily.

The standard charge sensitive preamplifier used here proves to be applicable, but crucial improvements in the electronic noise performance are still possible and necessary for the intended application. The development of a voltage preamplifier without feedback components which are not necessary for the sinusoidal ICD signals, is underway. In addition, the capacitance of the electrode structure to ground needs to be as low as possible for optimal SNR^34^.

The detection and false positive rate measurements show that even for a signal very close to noise level, a threshold can be found to make detections in real time and exclude false positives with >99.9% probability. This threshold can be higher than the pure ICD signal, because the measurement contains the sum of signal and noise. At a low signal-to-noise ratio, there is a considerable probability that a component of the total noise spectrum contributes at the same frequency as the expected signal so that the sum of signal and noise surpasses the threshold, a phenomenon referred to as stochastic resonance^35^.

For the aim of deterministic ion implantation, it will be required to open the beam blanker within a short time in the range of a few s after ion detection in the ICD. The real time signal analysis demonstrated here uses a modern oscilloscope able to produce a 5 V trigger output signal in response to certain trigger conditions, useful for such purposes (delay time: manufacturer^36^ 800 ns, measured 802.9 ± 0.1 ns). This time delay is compatible with the flight time of the ion from the ICD to the beam blanker.

For the envisaged single ion detection setup, it is planned to use ions charged up to much more than one elementary charge, if necessary, substantially increasing the signal strength above the noise floor. Especially, heavy ions like arsenic, antimony and bismuth are becoming interesting for silicon quantum technologies^14^. Even if they are prepared in high charge states, their velocity is not much higher than that of light-weight ions for the same acceleration potential. Furthermore, straggling is reduced for higher ion masses, allowing for higher spatial precision of the ion implant^14^.

Using Eq. 4, a figure of merit for a dedicated voltage preamplifier for single ion detection is developed. State-of-the-art input transistors can be optimised with an input capacitance of, e.g. 0.5 pF and a total input noise of $0.3\,{\rm{nV}}/\sqrt{{\rm{Hz}}}$. Suppose, the whole image charge detector is split into individual segments, each connected to a transistor and preamplifier in order to reduce the input capacitance of each preamplifier. The outputs of the preamplifiers are then combined again. The electrodes connected to one transistor then exhibit a typical capacitance of 0.5 pF and the time-of-flight of one ion through the whole detector array is approximately 1 s. For an *n* = 5 times charged ion *V*_peak_ = *ne*/*C*_total_ is ≈800 nV and the SNR as in Eq. 7 equates to

8 $${\rm{SNR}}=\frac{800\,{\rm{nV}}}{\sqrt{1\,{\rm{MHz}}}\cdot 0.3\,{\rm{nV}}/\sqrt{{\rm{Hz}}}}=\frac{8}{3}\mathrm{.}$$

These realistic values yield a SNR close to three which is sufficient for the envisaged detection, as the results above show. It is stressed, that this realistic opportunity of ion detection for deterministic implantation is enabled only by the specific situation of ion pre-detection with a detection probability allowed to be smaller than one. If the task were to achieve a detection rate of 100% or measure unknown properties of an ion, the SNR would be insufficient.

To estimate the maximum possible single ion implantation rate, the rate of ions arriving at the detector and the detection rate are limiting factors. It should be avoided that two or more ions are inside the detector at the same time. If the time needed to traverse the detector is in the order of 1 µs, the probability for more than one ion arriving within that time can be calculated from the Poisson distribution and a given rate of ions. For 1 × 10^5^ ions/s from the source, this probability is less than 0.01. With a detection rate of only 10%, about 1 × 10^4^ implanted ions per second is well achievable. Electrical switching times for beam blanking and scanning etc. should not impose any limitations in the microsecond regime.

## Conclusion

With this work, the state-of-the-art of image charge measurements is expanded towards application and optimisation of the ICD principle to the detection of a small number of low energy ions with the ultimate goal of single ion detecton for deterministic ion implantation in future. The central aspect is the fly-by detection during a single pass, including signal analysis within a limited time in the range smaller than 2 µs.

An image charge detection system has been built from scratch, deploying readily available and specifically designed electronics. Calibration and time-of-flight measurements were compared to theoretical considerations. Further, it was demonstrated how by using a linear array of ICD electrodes, the real time data analysis of a single pass of an ion bunch can be transferred to the frequency domain, which is a significant ingredient for future single ion detection. A simple detection threshold can be found to exclude false positive detections with a probability close to one, while the detection rate is still above 25% for this critically low SNR.

Work in the immediate future includes electrical simulations and experimental implementation of optimised electronics to lower the real time detection limit in the frequency domain below 10 elementary charges. Once again it is stressed, that the huge advantage of this approach to deterministic ion implantation is the possibility of discarding a certain fraction of ions from the source, allowing false positive detections to be sufficiently improbable. Live FFT, lock-in amplification or other algorithms implemented with dedicated electronics are tools that can be used to recover single ion fly-by signals in the frequency domain in real time, because the frequency of the signal is fixed and known.

In addition to showing the feasibility and requirements of deterministic ion implantation via image charge detection the knowledge gained is believed to be useful for the diagnostics and analysis of low current charged particle beams.

## Electronic supplementary material

Supplementary Material
