# Supplementary material for: Detection of small bunches of ions using image charges
Source: Sci Rep. 2018 Jun 28;8:9781. doi: 10.1038/s41598-018-28167-6 (PMC6023920; doi:10.1038/s41598-018-28167-6)
Supplement: Supplementary file 1 — Supplementary Material [file 41598_2018_28167_MOESM1_ESM.pdf]

Supplementary material: Detection of small bunches of ions using image charges

Paul Räcke[1,2,*], Daniel Spemann[2,3], Jürgen W. Gerlach[2,3], Bernd Rauschenbach[1,2,3], and Jan Meijer[1,2]

[1]Universität Leipzig, Felix Bloch Institute for Solid State Physics, Linnéstr. 5, 04103 Leipzig, Germany
[2]Leibniz Joint Lab "Single Ion Implantation", Permoserstr. 15, 04318 Leipzig, Germany
[3]Leibniz Institute of Surface Engineering (IOM), Permoserstr. 15, 04318 Leipzig, Germany
[*]paul.raecke@physik.uni-leipzig.de

Noise performance of the ICD1 set-up

The ICD1 set-up is the predecessor to the ICD2 set-up discussed in the main text. In ICD1, the vacuum-compatible Amptek A250 preamplifier is used on a printed circuit board (PCB) based on the A250 test board available from Amptek, with all parts assembled and soldered in our labs. The feedback capacitance is $C_\mathrm{f} = 1\,\mathrm{pF}$, the feedback resistance $R_\mathrm{f} = 300\,\mathrm{M\Omega}$. A photograph of the set-up is shown in Fig. 1. For comparison, the assembly of the ICD2 electrode array is shown as well. Note that the preamplifier electronics of ICD2 is placed outside the chamber and, therefore, not displayed in Fig. 1.

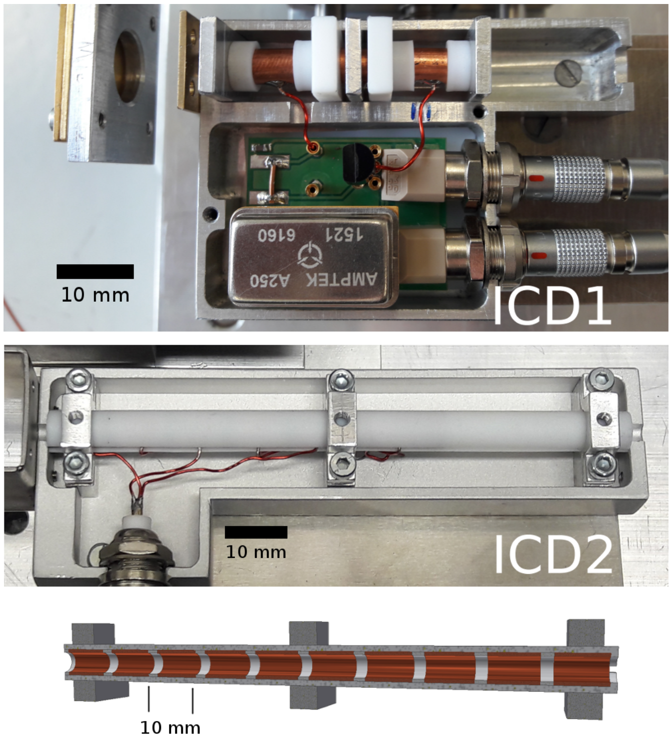

Figure 1. Photographs of the ICD1 set-up including preamplifier and the ICD2 eletrode array assembly. At the bottom a cross section of the ICD2 electrode array is sketched, in this case for ICD2.5.

As the preamplifier electronics of ICD1 is placed directly next to the electrode arrangement, the electric connections from the signal electrodes to the preamplifier input can be held as short as possible. Nevertheless, the ICD1 set-up is more susceptible to noise originating from sources outside (interferences through the power supply) and inside the vacuum chamber (beam blanking system) than the ICD2 set-up. This is illustrated in the inset of Fig. 2, where the interferences from switching the beam blanker for ion bunch formation can be seen at $t = -1.0\,\mu\mathrm{s}$ and $t = 0\,\mu\mathrm{s}$. These interferences can be suppressed by applying a

low-pass digital filter of suitable cut-off frequency. As Fig. 3 shows, the total noise is dominated by a non-statistical source, primarily due to interferences from radio signals, etc. from outside the chamber, because the averaging of N single acquisitions does not reduce the root-mean-square (rms) noise by $1/\sqrt{N}$ as expected for pure statistical uncorrelated noise. This is most prominent for the full bandwidth measurements. As these noise contributions mostly have frequencies much higher than the ICD signals itself, digital filtering can be effectively applied to improve the situation.

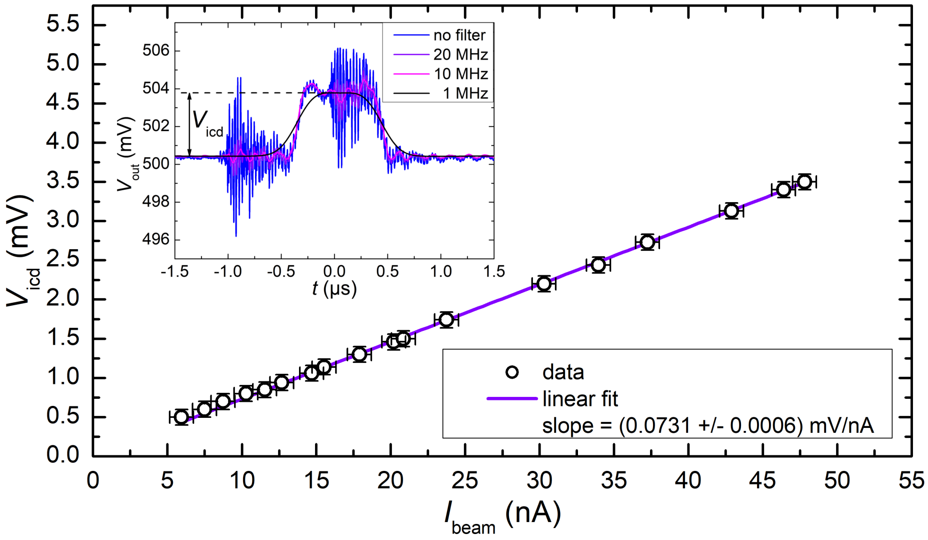

Figure 2. Linear ion beam current calibration curve for a 16 mm electrode in ICD1. The inset shows a typical pulse waveform for $t_{pulse} > t_E$ measured with different digital low-pass filter cut-off frequencies.

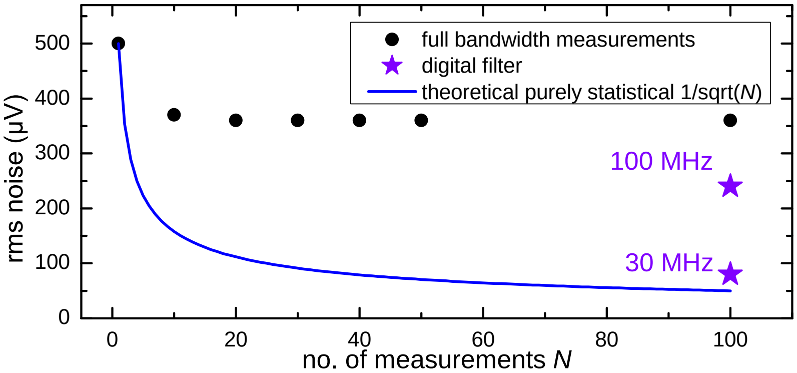

Figure 3. Noise measurements for ICD1 with two 8 mm signal electrodes, excluding the interference from switching the beam blanker, together with the calculated $1/\sqrt{N}$-dependence expected for pure statistical noise. If a low-pass digital filter with a cut-off frequency of 30 MHz is applied to the data prior averaging, the rms noise can be reduced almost to levels expected for pure statistical noise for $N = 100$.

Sensitivity calibration of and time-of-flight measurements with ICD1

The set-up ICD1 was used for calibration measurements, similar to those described for ICD2 with a single signal electrode. Here, a signal electrode of 16 mm length as employed and a sensitivity of $S = 0.63\,\mathrm{V/pC}$ achieved (see Fig 2).
Furthermore, the ICD1 set-up was used for time-of-flight measurements. For this purpose, two signal electrodes were employed, separated by insulating spacers and a grounded electrode. Fig. 4(a) shows the weighting potential distribution of this configuration simulated with SIMION. For the measurements, bunches formed from ion beams with different kinetic energies and ion species were used. Consequently, the detector output shows two peaks in the time domain as shown in Fig. 4(b) exemplarily for 5 keV Argon ion bunches. The time-of-flight was determined from the separation of the two peaks and is

compared in Table 1 to theoretical calculations of the time needed for the ions to travel the distance of 28 mm between the centres of the two signal electrodes. As can be seen, they are in very good agreement.

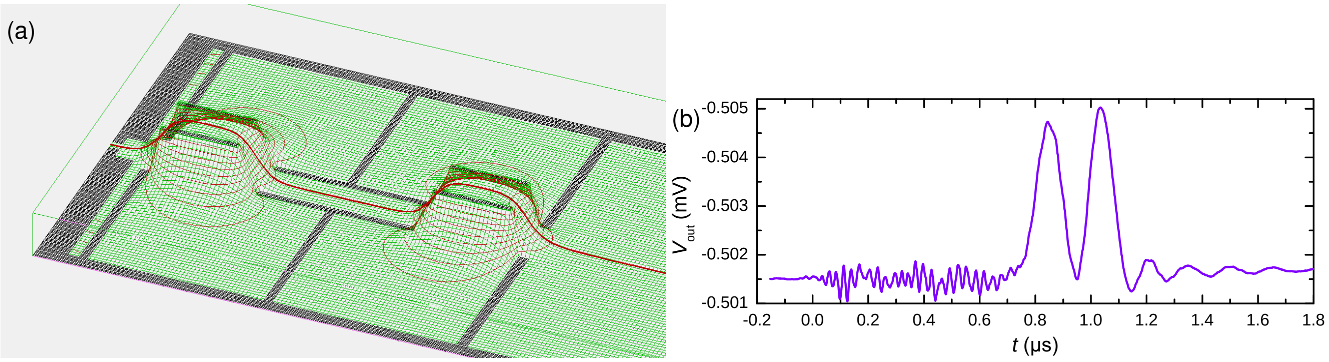

Figure 4. **(a)** SIMION simulation of the weighting potential landscape (green surface) on a section across the symmetry axis of the detector, for two 8 mm signal electrodes, separated by a 16 mm grounded electrode in ICD1. The grey lines represent the locations and geometry of the electrodes and conducting surfaces in this cross-section of the simulated arrangement. **(b)** Detector output signal averaged over 100 single digitally filtered acquisitions from 5 keV Argon ion bunches formed from a 62 nA ion beam, recorded with the same electrode arrangement as in (a).

	time-of-flight	
	experimental (ns)	theoretical (ns)
Ar^+ 5 keV	190 ± 10	181
Ar^+ 3 keV	230 ± 10	233
N_2^+ 5 keV	160 ± 10	151

Table 1. Comparison of the measured and calculated time-of-flight of different ion bunches for 28 mm distance of travel.
